# A novel mutation in STK11 gene is associated with Peutz-Jeghers Syndrome in Chinese patients

**DOI:** 10.1186/1471-2350-12-161

**Published:** 2011-12-14

**Authors:** Zhiqing Wang, Yulan Chen, Baoping Wu, Haoxuan Zheng, Jiman He, Bo Jiang

**Affiliations:** 1 Guangdong Provincial Key Laboratory of Gastroenterology, Department of Gastroenterology, Nanfang Hospital, Southern Medical University, Guangzhou 510515, China

## Abstract

**Background:**

Peutz-Jeghers syndrome (PJS) is caused by mutations in the tumor suppressor gene, STK11, and is characterized by gastrointestinal hamartomas, melanin spots on the lips, and an increased risk of developing cancer.

**Methods:**

Blood samples were collected from two unrelated Chinese PJS families totaling 20 individuals (9 male and 11 females), including 6 PJS patients. The entire coding region of the STK11 gene was amplified by polymerase chain reaction and analyzed by direct sequencing.

**Results:**

A novel mutation, c.904C > T, in exon 7 was identified in both families. A C > T substitution changed codon 302 from CAG (glutamine) to TAG (stop), truncating the STK11 protein, thus leading to the partial loss of the kinase domain and complete loss of the α-helix C-terminus. Furthermore, one PJS patient from each family was diagnosed with a visceral cancer, a colon cancer and a liver cancer respectively.

**Conclusion:**

We predict that this novel mutation, p.Q302X, is most likely responsible for development of the PJS phenotype and may even contribute to malignancy.

## Background

Peutz-Jeghers Syndrome (PJS; MIM#175200) is a rare autosomal dominant disorder characterized by gastrointestinal hamartomatous polyps, mucocutaneous pigmentation, and an increased risk for the development of gastrointestinal (GI) and various extra-GI malignancies [1,2]. The relative cancer risk has been estimated to be 9-18 times higher in PJS patients than the general population [3,4].

Mutations in the serine-threonine kinase 11 (STK11/LKB1, MIM#602216) gene on chromosome 19p13.3 have been considered to be the major cause of PJS [5,6]. The gene is divided into nine exons that encode a 433 amino acid protein, which acts as a tumour suppressor. Mutation detection rates of 10-94% have been achieved at different centers, depending on the screening method, with considerable uncharacterized genetic heterogeneity remaining in this syndrome [2,7]. Most mutations are frameshift or nonsense changes, which result in an abnormal truncated protein and the consequent loss of kinase activity [8]. However, the STK11 mutation spectrum and genotype-phenotype correlation are still poorly understood.

Here, we report a novel nonsense mutation, c.904C > T (p.Q302X), in exon 7 from two unrelated Chinese PJS families that subsequently developed colon cancer and liver cancer, respectively.

## Methods

### Family study

**Figure 1:**
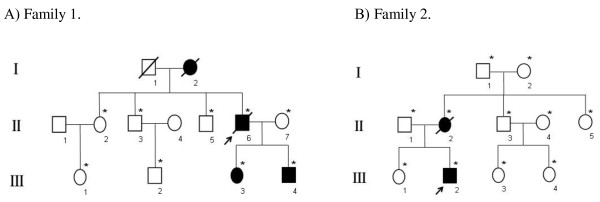
**Pedigree of the families with PJS**. Roman numerals indicate generations and arabic numbers indicate individuals. Squares = males, circles = females. Affected individuals are denoted by solid symbols and unaffected individuals are denoted by open symbols. A slash denotes that the individual is deceased. The initial proband is indicated by an arrow, and participants in the DNA analysis are marked with an asterisk.

Family 1, from Guangdong province, China, consisted of three generations affected with PJS, including two female and two male individuals (Figure 1A). The proband (II:6) died from colon adenocarcinoma at 61 years of age. Family 2, from Hongkong, China, included two PJS patients (Figure 1B). The proband (III:2) in that family underwent abdominal surgery three times due to intussusception and obstruction of the small intestine from Peutz-Jeghers polyps when he was 16, 23, and 25 years old. The mucocutaneous pigmentation faded gradually after his third surgery, but was still visible on his lips and buccal mucosa at the time of this study. His mother (II:2) presented no obvious symptoms until the time of liver cancer diagnosis at 45 years of age. The clinical features of the affected individuals are summarized in Table 1.

**Table 1:** Characteristics of affected individuals in two Chinese PJS families.

Individual	Family 1				Family 2	
	I : 2	II : 6	III : 3	III : 4	II : 2	III : 2
**Sex**	F	M	F	M	F	M
**Age at onset of mucocutaneous pigmentations**	unknown	2 years	3 years	3 years	6 months	First year
**Age at onset abdominal symptoms**	34	22	14	4	No	15
Age*	34*	61*	27	23	45*	26
**Anemia**	unknown	Yes	Yes	Yes	No	Yes
**Rectal bleeding**	unknown	Yes	Yes	Yes	No	Yes
**Rectal prolapse**	unknown	Yes	Yes	No	No	Yes
**Number of operations for intussusception (Age)**	0	1 22 y	1 15 y	1 19 y	0	3 16, 23, 25 y
**Cause of death**	Intussusception	Colon cancer	-	-	Liver cancer	-

The diagnostic criteria for PJS include the presence of characteristic mucocutaneous pigmentation, the presence of hamartomatous polyps in the gastrointestinal tract, and a family history of PJS. Patients need to fulfill two of these three criteria for a clinical diagnosis of the disease [9]. This study was approved by the Medical Ethics Committee, Nanfang Hospital of Southern Medical University, and informed consent was obtained from all participating individuals.

### Mutation detection

Genomic DNA was extracted from peripheral blood using the QiAamp DNA Blood Midi Kit (Qiagen, Hilden, Germany). PCR primers were designed by Primer 3.0 (http://frodo.wi.mit.edu/primer3/) to amplify the STK11 (GeneBank NM_000455) coding regions and intron-exon boundaries. Details of primer sequences and PCR conditions are available upon request. The PCR products were then sequenced in both directions on an ABI 3730 XL Automated DNA Sequencer (Applied Biosystems, Foster City, CA) with the ABI BigDye Terminator v3.1 cycle sequencing kit. Standard nomenclature (http://www.hgvs.org/mutnomen/) was used to describe sequence variations, with +1 corresponding to the A of the ATG translation initiation codon of GeneBank NM_000455 for STK11.

All patients and available family members underwent STK11 germline mutation testing to confirm co-segregation of the mutation with the disease. In order to rule out polymorphisms and to confirm the pathogenic effects of variations, 100 chromosomes from 50 unrelated healthy individuals were also screened for the presence of the mutation.

### Structural predictions of the wide-type and mutant STK11 protein

The homology modeling programs, SWISS MODEL (http://swissmodel.expasy.org//SWISS-MODEL.html) and CPH models, (http://www.cbs.dtu.dk/services/CPHmodels/) were used to develop an appropriate model to mimic the effects of the mutated region.

## Results

**Figure 2:**
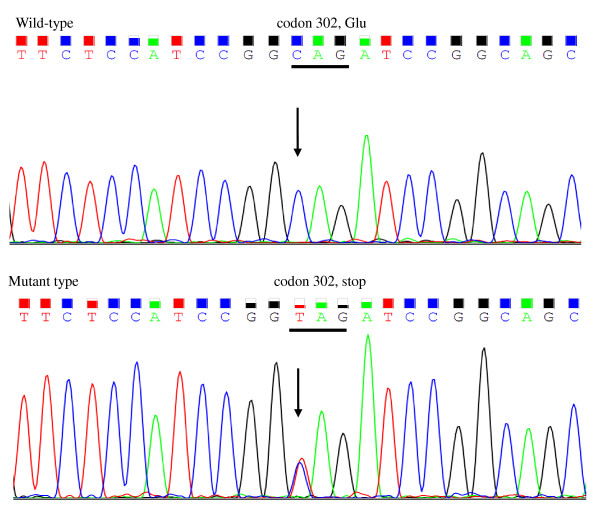
**The germline nonsense mutation of the STK11 gene**. Arrows indicate the position of the mutation and the underlines highlight the codon containing the mutation. The wide-type sequence CAG (glutamine) is altered to the mutant sequence TAG (stop) at codon 302, which leads to truncation of the STK11 protein.

**Figure 3:**
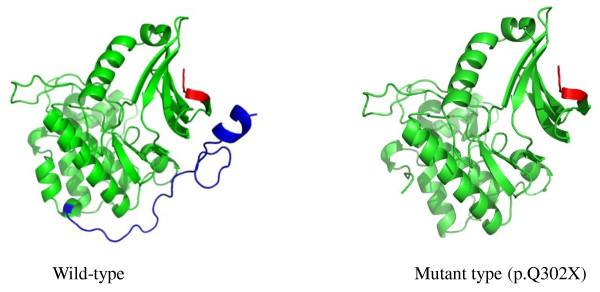
**Structural modeling of the wild-type and mutant proteins**. The STK11 protein is mainly comprised of three major domains: the N-terminal non-catalytic domain in red, the catalytic kinase domain in green, and the C-terminal non-catalytic regulatory domain in blue. The mutation, p.Q302X, leads to partial loss of the kinase domain and complete loss of the C-terminal end of the α-helix.

Direct sequencing of the STK11 gene showed a *C-to-T *transversion of nucleotide 904 in exon 7 in these two unrelated families, which altered the wild-type sequence CAG (glutamine) to the mutant sequence TAG (stop) at codon 302, and resulted in a truncation of the STK11 protein (Figure 2). Our structural prediction revealed that this mutation leads to partial loss of the kinase domain and complete loss of the C-terminal end of the α-helix (Figure 3). This mutation has not yet been described in the literature.

All affected patients carried this novel mutation, but none of their unaffected relatives or normal controls had the mutation. Since the mutation clearly co-segregated with the disease phenotype in these two families, and the mutation was predicted to affect the expression of the STK11 gene product, we concluded that this mutation is disease-specific for these kindreds and not a polymorphic variant of the STK11 gene. Both families are from the southern part of China, and therefore it is probable that this mutation has a common origin, even though the two families were screened at different centers.

## Discussion

The STK11 protein is mainly comprised of three major domains: an N-terminal non-catalytic domain that contains the nuclear localization signal, a catalytic kinase domain that is important for ATP binding, and a C-terminal non-catalytic regulatory domain that contains a prenylation motif (CAAX-box) [10]. Codons 49-309 encode the catalytic kinase domain. The C-terminal non-catalytic region of the STK11 protein is encoded by exon 8 and 9, and encompasses amino acids 309-433. In these two Chinese families, the mutation, c.904C > T (p.Q302X), results in the premature termination of the 433 amino acid protein at codon 302, which leads to partial loss of the kinase domain and complete loss of the C-terminal regulatory domain (CRD). With this mutation, patient II:6 in family 1 developed colon adenocarcinoma at 60 years of age and died one year later, and patient II:2 in family 2 died from liver cancer at 45 years of age. These findings suggest that the novel mutation, p.Q302X, is most likely responsible for the development of the PJS phenotype and may even contribute to malignancy.

Although the exact function of STK11 remains largely unknown, studies suggest that it plays a role in cell signaling and apoptosis [11]. Development of the PJS phenotypes is believed to be due to the elimination of the kinase activity of STK11, which is associated with a loss of growth suppression function [12]. Recently, several proteins, including p53, Cdc37, Hsp90, and PTEN, which are responsible for cancer syndromes when mutated or involved in cancer pathways, were reported to interact with the kinase domain of STK11; however, the protein interaction domains have not been mapped [13]. Accordingly, the interaction domain for one of these proteins may be localized to codon 302, and therefore could explain the high cancer risk of mutations within this site. Moreover, the C-terminal domain of STK11 is important for binding STRAD, which is a protein that is possibly involved in MAPK signaling, control of the AMPK pathway (a key regulator of cellular metabolism), and control of cell polarity [14,15]. Taken together, these data suggest that the novel STK11 mutation, c.904C > T (p.Q302X), which was found in two unrelated Chinese PJS families, causes partial loss of the kinase domain and complete loss of the C-terminal domain, and may contribute to polyp formation and tumorgenesis through various mechanisms, such as loss of growth arrest, apoptosis, and loss of cell polarity. Additional studies are needed to address these questions.

A genotype-phenotype correlation has been sought in PJS. Schumacher *et al. *[16] suggested that in-frame mutations in the domains that encode the protein and ATP binding and catalysis (I-VIA) are rarely associated with cancer. Moreover, missense mutations in the C-terminus and in the domains for substrate recognition (VIB-VIII) seem to be more associated with malignancies, and patients with breast carcinomas predominantly had truncating mutations. Mehenni *et al. *[13] suggested that there was a higher risk of cancer in cases with mutations in exon 6 of the STK11 gene. In a large analysis of 240 PJS patients with STK11 mutations, no difference was observed between individuals with missense and truncating mutations or between familial and sporadic cases, although it was suggested that there was a higher risk of cancer in individuals with mutations in exon 3 of the gene. Hearle *et al. *[2] extended this study by analyzing a total of 419 PJS patients, of whom 297 had documented mutations. In that study, it was found that the type and site of mutation did not influence cancer risk.

## Conclusion

In our current study, we identified the novel mutation, p.Q302X, which was associated with cancers in both Chinese families where it was identified. Members of PJS families are at risk of developing the disease. Identification of a STK11 gene mutation in an index patient offers the possibility of a predictive diagnosis in PJS pedigrees. Furthermore, a possible genotype-phenotype correlation, especially concerning the risk of gastrointestinal and extraintestinal cancers, would be of particular clinical interest.

## Abbreviations

PJS: Peutz-Jeghers syndrome; STK11: Serine threonine kinase 11; GI: gastrointestinal; CRD: C-terminal regulatory domain; Cdc37: Cell division cycle 37; Hsp90: Heat shock protein; PTEN: phosphatase and tensin homolog deleted on chromosome ten; STRAD: STE20-related adaptor; MAPK: mitogen-activated protein kinase; AMPK: AMP-activated protein kinase.

## Competing interests

The authors declare that they have no competing interests.

## Authors' contributions

ZQW carried out molecular genetic studies including sequencing for all the families and the controls, and drafted the manuscript. YLC did the structural predictions. BPW identified and diagnosed the patients. HXZ and JMH performed GI examinations and provided histopathological information. BJ designed, supervised the study and revised the manuscript. All authors read and approved the final manuscript.

## Pre-publication history

The pre-publication history for this paper can be accessed here:

http://www.biomedcentral.com/1471-2350/12/161/prepub
